# Machine learning-based preoperative analytics for the prediction of anastomotic leakage in colorectal surgery: a swiss pilot study

**DOI:** 10.1007/s00464-024-10926-4

**Published:** 2024-05-22

**Authors:** Stephanie Taha-Mehlitz, Larissa Wentzler, Fiorenzo Angehrn, Ahmad Hendie, Vincent Ochs, Julia Wolleb, Victor E. Staartjes, Bassey Enodien, Martinas Baltuonis, Stephan Vorburger, Daniel M. Frey, Robert Rosenberg, Markus von Flüe, Beat Müller-Stich, Philippe C. Cattin, Anas Taha, Daniel Steinemann

**Affiliations:** 1 Clarunis, University Center for Gastrointestinal and Liver Diseases, St. Clara Hospital and University Hospital Basel, 4002 Basel, Switzerland; 2 Medical Faculty, University Basel, 4056 Basel, Switzerland; 3 Center for Gastrointestinal and Liver Diseases, Cantonal Hospital Basel-Landschaft, 4410 Liestal, Switzerland; 4 Department of Computer Engineering, McGill University, Montreal, H3A 0E9 Canada; 5 Department of Biomedical Engineering, Faculty of Medicine, University of Basel, Hegenheimermattweg 167C Allschwil, 4123 Basel, Switzerland; 6 Machine Intelligence in Clinical Neuroscience (MICN) Laboratory, Department of Neurosurgery, University Hospital Zurich, 8091 Zurich, Switzerland; 7 Department of Surgery, GZO-Hospital, 8620 Wetzikon, Switzerland; 8 Department of Surgery, Emmental Teaching Hospital, 3400 Burgdorf, Switzerland; 9 Hirslanden Klinik St. Anna, 6006 Lucerne, Switzerland; 10 Department of Surgery, Brody School of Medicine, East Carolina University, Greenville, NC USA

**Keywords:** Anastomotic insufficiency, Anastomotic leakage, Machine learning, Colorectal surgery, Prediction tool, Prediction of anastomotic leakage

## Abstract

**Background:**

Anastomotic leakage (AL), a severe complication following colorectal surgery, arises from defects at the anastomosis site. This study evaluates the feasibility of predicting AL using machine learning (ML) algorithms based on preoperative data.

**Methods:**

We retrospectively analyzed data including 21 predictors from patients undergoing colorectal surgery with bowel anastomosis at four Swiss hospitals. Several ML algorithms were applied for binary classification into AL or non-AL groups, utilizing a five-fold cross-validation strategy with a 90% training and 10% validation split. Additionally, a holdout test set from an external hospital was employed to assess the models' robustness in external validation.

**Results:**

Among 1244 patients, 112 (9.0%) suffered from AL. The Random Forest model showed an AUC-ROC of 0.78 (SD: ± 0.01) on the internal test set, which significantly decreased to 0.60 (SD: ± 0.05) on the external holdout test set comprising 198 patients, including 7 (3.5%) with AL. Conversely, the Logistic Regression model demonstrated more consistent AUC-ROC values of 0.69 (SD: ± 0.01) on the internal set and 0.61 (SD: ± 0.05) on the external set. Accuracy measures for Random Forest were 0.82 (SD: ± 0.04) internally and 0.87 (SD: ± 0.08) externally, while Logistic Regression achieved accuracies of 0.81 (SD: ± 0.10) and 0.88 (SD: ± 0.15). F1 Scores for Random Forest moved from 0.58 (SD: ± 0.03) internally to 0.51 (SD: ± 0.03) externally, with Logistic Regression maintaining more stable scores of 0.53 (SD: ± 0.04) and 0.51 (SD: ± 0.02).

**Conclusion:**

In this pilot study, we evaluated ML-based prediction models for AL post-colorectal surgery and identified ten patient-related risk factors associated with AL. Highlighting the need for multicenter data, external validation, and larger sample sizes, our findings emphasize the potential of ML in enhancing surgical outcomes and inform future development of a web-based application for broader clinical use.

Anastomotic Leakage (AL) is a severe and potentially deadly complication of significant clinical importance following gastrointestinal surgery [1]. According to Rahbari et al. [2], AL is defined as a defect at the anastomotic site that results in a connection between intra—and extraluminal compartments. AL is considered an independent risk factor for adverse clinical and oncological outcomes like decreased survival of cancer patients and increased readmission rates after surgery [3–5]. The approximated incidence of AL is 3.3% after colon anastomosis and 8.6% after colorectal anastomosis in specialized centers [4]. However, these rates are likely considerably higher in centers lacking dedicated colorectal surgery teams and after emergency surgery. In fact, depending on diagnostic criteria, AL rates of over 10% have been reported in the literature [6–10]. Hospital stay is extended by twelve days on average, and healthcare-related expenses are increased by up to 30.000 USD in patients who experience AL [1, 2].

In previous publications, a multitude of risk factors for AL have been identified more or less consistently, e.g., age, body mass index (BMI), comorbidity indexes, emergency surgery, steroids, or active smoking [6–25]. Integrating all of these risk factors into one holistic clinical prediction of AL is a very challenging task, even for experienced physicians. Indeed, even experienced surgeons were reported to systematically underestimate the risk of AL by clinical assessment [26]. Undoubtedly, the ability to preoperatively predict AL precisely would allow for better resource allocation, enhanced patient preparation, and improved patient–physician relationships due to the improved quality of informed consent. Specifically, by identifying preoperative risk factors on the one hand, the modifiable risk factors could be addressed to reduce the individual patient risk of AL. On the other hand, for such patients, modification of the surgical approach could be considered; for example, the creation of a deviating stoma to mitigate the consequences of AL.

Machine learning (ML) algorithms can be exceptionally competent at integrating diverse patient variables into a unified risk model that can generate predictions specific to each patient. However, the development and rigorous validation of clinical prediction models require large amounts of multicentre data as well as external validation. Before embarking on said multicenter data collection, piloting a modeling strategy to assess the feasibility and identify the most valuable inputs is crucial. Consequently, the aim of this pilot study is to assess whether AL can be predicted from preoperative data from four Swiss surgical centers using machine learning (ML) algorithms.

## Methods

### Overview and data collection

Data were extracted retrospectively from the patient registry of the University Hospital of Basel, the GZO Hospital Wetzikon, Emmental Teaching Hospital, and the Cantonal Hospital Liestal and entered into a shared REDCap database. The data collection was performed by consultants, surgical residents, or master students of the medical field under supervision. Patients who underwent colon anastomosis for various reasons, including neoplasia, diverticulitis, ischemia, iatrogenic or traumatic perforation, or inflammatory bowel disease between 1st of January 2012 and 31st of December 2020 and had a follow-up of at least 6 months were eligible where general consent was available. This study was completed based on the transparent reporting of a multivariable prediction model for individual prognosis or diagnosis (TRIPOD) statement checklist for the development of clinical prediction models [27]. Utilizing the aforementioned data, we developed ML models with the aim of predicting AL.

### Patient and public involvement

Patients and the public have not been involved in planning, managing, designing or carrying out the research.

### Predictors and outcome measures

AL was defined according to Gessler et al. [28] and Rahbari et al. [2] as any clinical sign of leakage, confirmed by radiological examination, endoscopy, clinical examination of the anastomosis, or upon reoperation. Recorded variables included 21 risk factors that already have been reported in the literature such as age, sex, body mass index (BMI), active smoking (up to 6 weeks before surgery), alcohol abuse (> 2 alcoholic beverages per day), prior abdominal surgery, preoperative leucocytosis (≥ 10.000 per mm^3^), preoperative steroid use, Charlson Comorbidity index (CCI), American Society of Anesthesiologists (ASA) score, renal function (*chronic kidney disease (*CKD) stages G1 to G5), albumin (g/dl), and hemoglobin level (g/dl), liver metastasis (at the time of surgery proven by radiological imaging or biopsy preoperatively), indication (e.g., tumor, diverticular disease, ileus, ischemia, inflammatory bowel disease), type of surgery (right or left sided hemicolectomy, ileocecal resection, transverse colectomy, sigmoidectomy, rectosigmoidectomy, colostomy, or Hartmann’s reversal), emergency surgery, bowel perforation, surgical approach (laparoscopic, robotic or open), anastomotic technique (hand-sewn or stapler), and defunctioning ileostomy.

### Model development

Python 3.10 was used to perform all analyses. The Sklearn and xgb packages were used for implementing all machine learning models, including Logistic Regression (generalize linear model; GLM), Lasso Regression (lasso), Artificial Neural Network, Random Forest, Extreme Gradient Boosting (XGBoost), and Support Vector Machine (SVM) models [29]. For data preprocessing, missing values were replaced by − 1 for both numeric and categorical features. For data normalization, one-hot encoding was used for categorical features and minmax scaling for numeric features [30]. To avoiding overfitting, data augmentation was used by applying Gaussian noise to the dataset to increase the number of samples synthetically [31].

To evaluate model performance, data were split into random test set (10%) and a train set (90%). In this study, we employed a five-fold cross-validation methodology for model training. A gridsearch algorithm based on the F1 Score was used to tune the hyperparameters. In addition, for testing the model’s robustness, an additional test set from another hospital has been used separately.

## Results

### Cohort

**Fig. 1:**
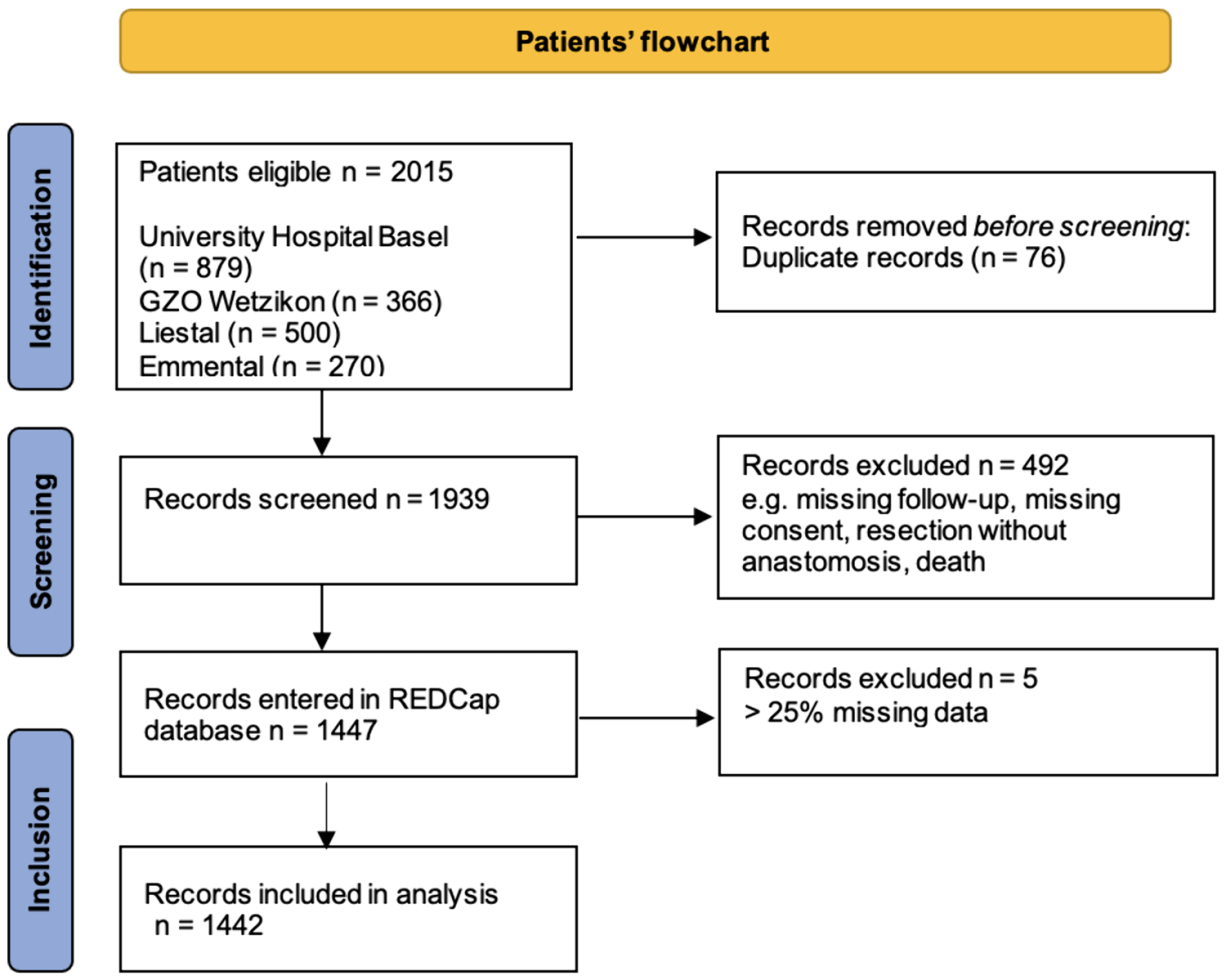
Flowchart of included patients from all hospitals

In the training process, a total of 1244 patients were included in the training set, of which 112 (9.0%) suffered from AL. Figure 1 shows the flowchart of the patients included into the study. Only patients were entered into the database where general consent was available. Other reasons why patients did not qualify for data entry were mostly missing follow-up, colonic resection without anastomosis, death after surgery, or a deviating stoma still in place at last follow-up. A total of 5 patients had missing data > 25% and thus were excluded from the algorithm. In the holdout test set, a total of 198 patients were included, of which 7 (3.5%) suffered from AL.

The mean age was 65.5 ± 14.8 (holdout test set: 64.7 ± 14.6). Furthermore, 578 (46.5%) patients were male and 666 (53.5%) female (holdout test set: 97 male [49%] and 101 female [51%]). The mean BMI was 25.7 ± 5.3 (holdout test set: 27.9 ± 5.5) and 292 (23.5%) patients were active smokers (holdout test set: 13 [6.6%]). Tables 1 and  2 provide an overview of the cohorts.

**Table 1:** Overview of patient characteristics for training data

Variable	Missing	Total *n* = 1244	AL *n* = 112	No AL *n* = 1132
Age (years)	0%	65.5 ± 14.8	65.9 ± 14.8	65.4 ± 14.8
Sex	0%	Female–666 (53.5%)	Female—51 (45.5%)	Female—615 (54.3%)
BMI (kg/m^2^)	6.3%	25.7 ± 5.3	26.2 ± 5.9	25.7 ± 5.2
Active smoking	8.1%	Yes—292 (23.5%)	Yes—43 (38.4%)	Yes—249 (22.0%)
Alcohol abuse	0.3%	Yes—113 (9.1%)	Yes—19 (17.0%)	Yes—94 (8.3%)
CCI	0%	4.4 ± 3.4	6.0 ± 4.1	4.2 ± 3.3
ASA	0.4%	1–16 (1.3%)	1–0 (0.0%)	1–16 (1.4%)
		2–436 (35.0%)	2–22 (19.6%)	2–414 (36.6%)
		3–692 (55.6%)	3–80 (71.4%)	3–612 (54.1%)
		4–90 (7.2%)	4–9 (8.1%)	4–81 (7.2%)
		5–5 (0.4%)	5–1 (0.9%)	5–4 (0.3%)
Renal function	3.4%	G1—427 (34.3%)	G1—37 (33.0%)	G1—390 (34.5%)
		G2—496 (39.9%)	G2—40 (35.7%)	G2—456 (40.3%)
		G3—244 (19.6%)	G3—25 (22.3%)	G3—219 (19.3%)
		G4—32 (2.6%)	G4—8 (7.1%)	G4—24 (2.1%)
		G5—3 (0.2%)	G5—0 (0.0%)	G5—3 (0.3%)
Albumin (g/dl)	31.3%	35.0 ± 7.8	31.1 ± 7.8	35.5 ± 7.7
Hemoglobin (g/dl)	3.2%	12.6 ± 2.1	12.2 ± 2.3	12.7 ± 2.1
Leukocytosis (≥ 10.000 per mm^3^)	3.3%	Yes—312 (25.0%)	Yes—40 (35.7%)	Yes—272 (24.0%)
Preoperative steroid use	0.2%	Yes—57 (4.6%)	Yes—13 (11.6%)	Yes—44 (3.9%)
Prior abdominal surgery	1.4%	Yes—583 (46.9%)	Yes—60 (53.6%)	Yes—523 (46.2%)
Liver metastasis	2%	Yes—73 (5.9%)	Yes—7 (6.3%)	Yes—66 (5.8%)
Indication	0%			
tumor		535 (43.0%)	51 (45.5%)	484 (42.8%)
IBD		30 (2.4%)	5 (4.5%)	25 (2.2%)
diverticular disease		424 (34.1%)	31 (27.7%)	393 (34.7%)
ischemia/ileus		72 (5.8%)	9 (8.0%)	63 (5.6%)
other		183 (14.7%)	16 (14.3%)	167 (14.8%)
Perforation	0%	Yes—156 (14.4%)	Yes—22 (19.6%)	Yes—145 (12.8%)
Emergency surgery	0%	Yes—257 (23.8%)	Yes—50 (44.6%)	Yes—262 (23.1%)
Surgical procedure	0%			
(extended) left hemicolectomy		95 (7.6%)	11 (9.8%)	84 (7.4%)
(extended) right hemicolectomy		333 (26.8%)	35 (31.3%)	298 (26.3%)
ileocecal resection		101 (8.1%)	12 (10.7%)	89 (7.9%)
transverse colectomy		25 (2.0%)	2 (1.8%)	23 (2.0%)
rectosigmoid resection/sigmoidectomy		628 (50.5%)	46 (41.1%)	582 (51.4%)
Hartmann’s reversal or reversal of colostomy		62 (5.0%)	6 (5.3%)	56 (4.9%)
Laparascopic	0.2%	Yes—707 (56.8%)	Yes—47 (42.0%)	Yes—660 (58.3%)
Technique (hand-sewn)	2.7%	Yes—505 (40.6%)	Yes—49 (43.8%)	Yes—456 (40.3%)
Defunctioning ileostomy	0.2%	Yes—119 (9.6%)	Yes—21 (18.8%)	Yes—98 (8.7%)

Absolute numbers and percentages for categorical or mean ± SD for continuous variables are presented

*BMI* Body mass index *CCI* charlson comorbidity index, *ASA* American society of anesthesiologists score, *IBD* inflammatory bowel disease

**Table 2:** Overview of patient characteristics for external validation data

Variable	Missing	Total *n* = 198	AL *n* = 7	No AL *n* = 191
Age (years)	0%	64.7 ± 14.6	71.7 ± 10.2	64.4 ± 14.7
Sex	0%	Female—101 (51.0%)	Female—4 (57.1%)	Female—97 (50.1%)
BMI (kg/m^2^)	1%	27.9 ± 5.5	28.2 ± 5.6	27.9 ± 4.6
Active smoking	1.5%	Yes—13 (6.6%)	Yes—1 (14.3%)	Yes—12 (6.3%)
Alcohol abuse	0.5%	Yes—1 (0.5%)	Yes—0 (0%)	Yes—1 (0.5%)
CCI	0%	3.9 ± 3.0	5.1 ± 2.9	3.9 ± 3.0
ASA	0%	1–17 (8.6%)	1–0 (0%)	1–17 (8.9%)
		2–147 (74.2%)	2–6 (85.7%)	2–141 (73.8%)
		3–34 (17.2%)	3–1 (14.3%)	3–33 (17.3%)
		4–0 (0%)	4–0 (0%)	4–0 (0%)
		5–0 (0%)	5–0 (0%)	5–0 (0%)
Renal function	0%	G1—175 (88.3%)	G1—6 (85.7%)	G1—69 (88.5%)
		G2—10 (5.1%)	G2—0 (0%)	G2—10 (5.2%)
		G3—13 (6.6%)	G3—1 (14.3%)	G3—12 (6.3%)
		G4—0 (0%)	G4—0 (0%)	G4—0 (0%)
		G5—0 (0%)	G5—0 (0%)	G5—0 (0%)
Albumin (g/dl)	96.5%	39.3 ± 10.1	–	39.3 ± 10.1
Hemoglobin (g/dl)	2.5%	13.8 ± 1.6	12.5 ± 0.7	13.8 ± 1.6
Leukocytosis (≥ 10.000 per mm^3^)	2.0%	Yes—44 (22.2%)	Yes—2 (28.6%)	Yes—42 (22.0%)
Preoperative steroid use	0.5%	Yes—6 (3.0%)	Yes—0 (0%)	Yes—6 (3.1%)
Prior abdominal surgery	5.6%	Yes—86 (43.4%)	Yes—4 (57.1%)	Yes—82 (42.9%)
Liver metastasis	2.5%	Yes—7 (3.5%)	Yes—0 (0%)	Yes—7 (3.7%)
Indication	0%			
tumor		93 (47.0%)	3 (42.3%)	90 (47.1%)
IBD		7 (3.5%)	1 (14.3%)	6 (3.1%)
diverticular disease		85 (42.9%)	0 (0%)	85 (44.5%)
ischemia/ileus		5 (2.5%)	1 (14.3%)	4 (2.1%)
other		8 (4.0%)	2 (28.6%)	6 (3.1%)
Perforation	0.5%	Yes—20 (10.1%)	Yes—4 (57.1%)	Yes—16 (8.4%)
Emergency surgery	0%	Yes—29 (14.6%)	Yes—3 (42.3%)	Yes—26 (13.6%)
Surgical procedure	0%			
(extended) left hemicolectomy		29 (14.6%)	1 (14.3%)	28 (14.7%)
(extended) right hemicolectomy		59 (30.0%)	3 (42.3%)	56 (29.3%)
ileocecal resection		7 (3.5%)	2 (28.6%)	5 (2.6%)
transverse colectomy		2 (1.0%)	0 (0%)	2 (1.0%)
rectosigmoid resection/sigmoidectomy		101 (51.0%)	1 (14.3%)	100 (52.4%)
Hartmann’s reversal or reversal of colostomy		0 (0%)	0 (0%)	0 (0%)
Laparascopic	0%	Yes—158 (84.8%)	Yes—3 (42.0%)	Yes—165 (86.4%)
Technique (hand-sewn)	0%	Yes—19 (9.6%)	Yes—0 (0%)	Yes—19 (10.0%)
Defunctioning ileostomy	0%	Yes—5 (2.5%)	Yes—1 (14.3%)	Yes—4 (2.1%)

Absolute numbers and percentages for categorical or mean ± SD for continuous variables are presented

*BMI* Body mass index, *CCI* charlson comorbidity index, *ASA* American society of anesthesiologists score, *IBD* inflammatory bowel disease

### Model performance

**Fig. 2:**
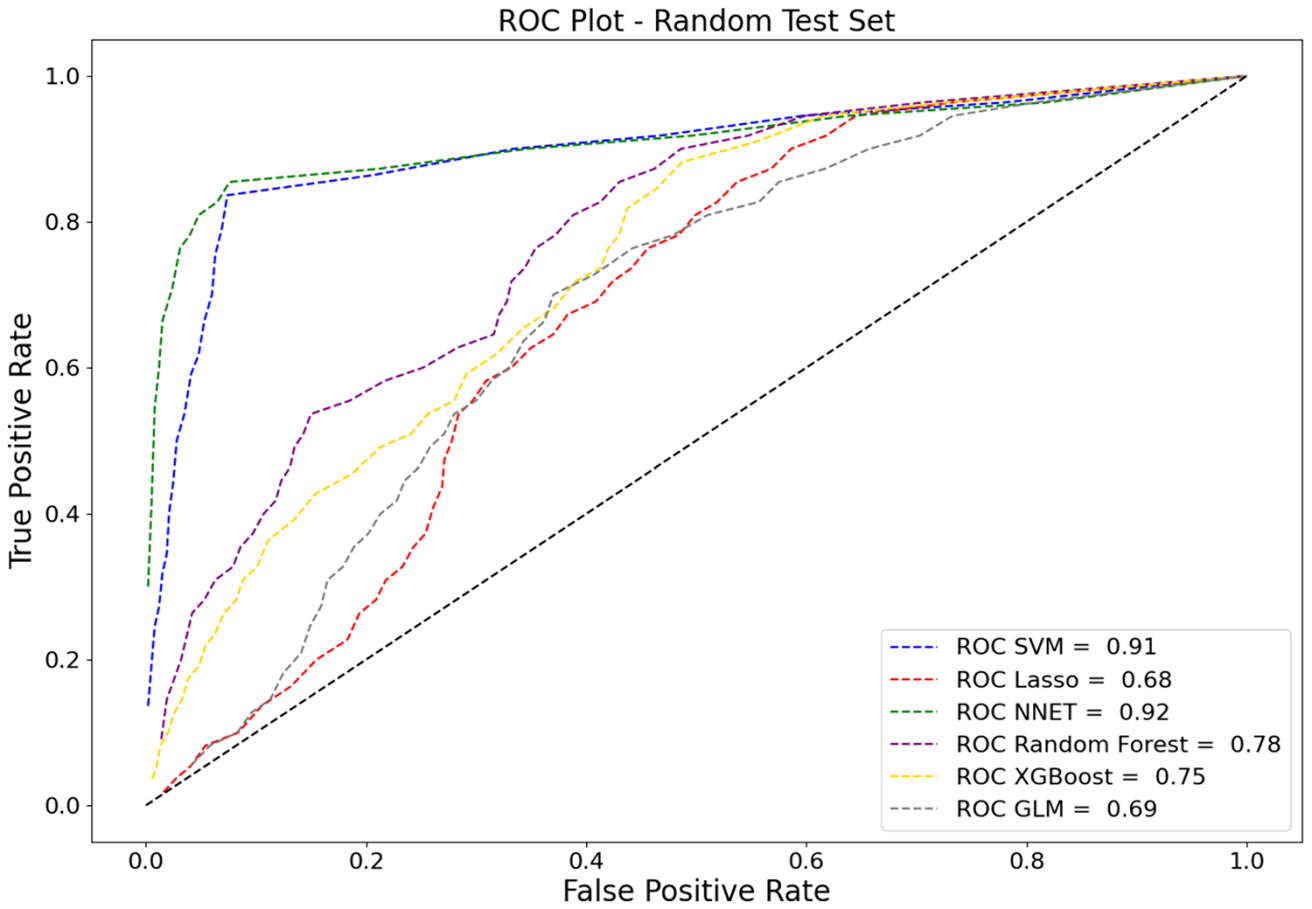
Area under the receiver operating characteristics curves (ROC-AUC) of the implemented models on the random test set

**Fig. 3:**
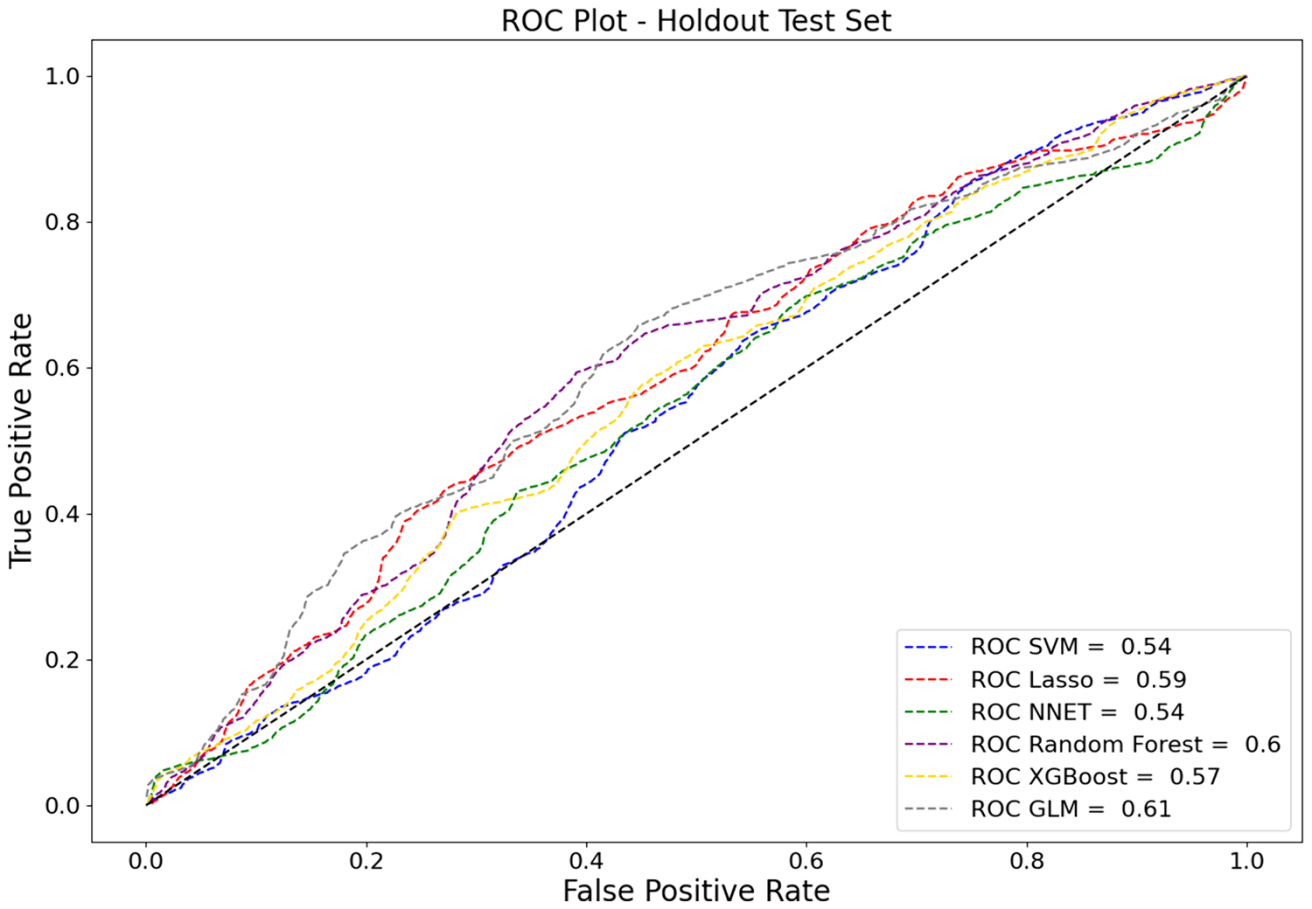
Area under the receiver operating characteristics curves (ROC-AUC) of the implemented models on the external holdout test set *GLM* Logistic regression, *lasso* lasso regression, *NNET* artificial neural network, *RF* random forest classifier, *SVM* support vector machine, XGBoost extreme gradient boosting

The Random Forest model demonstrated a good performance for binary classification, with an area under the receiver operating characteristic (AUC) of 0.78 (SD: [± 0.01]) and an accuracy of 0.82 (SD: [± 0.04]). Additionally, it achieved an F1 Score of 0.58 (SD: [± 0.03]). The model’s performance on the external holdout test set achieved an AUC score of 0.60 (SD: [± 0.05]), an accuracy of 0.87 (SD: [± 0.08]), and a F1 Score of 0.51 (SD: [± 0.03]). Considering the performance for the out-of-domain generalization, the Logistic Regression model performed with an AUC of 0.69 (SD: [± 0.01]) on the random test set and 0.61 (SD: [± 0.05]) on the holdout test set, and with an F1 Scores of 0.53 (SD: [± 0.04]) and 0.51 (SD: [± 0.02]), respectively. The performance of other models and additional metrics are detailed in Table 3. Specific feature importance within the models is highlighted in Table 4. Comparative ROC-AUC curves for the models, illustrating their performance in the random test set and the holdout test set, are presented in Fig. 2 and Fig. 3, respectively.

**Table 3:** Performance evaluation of all models used

Model	Metric	Results random test set	Results holdout test set
Logistic regression	Accuracy	0.81 ± 0.10	0.88 ± 0.15
	AUC	0.69 ± 0.01	0.61 ± 0.05
	Precision	0.25 ± 0.18	0.09 ± 0.10
	Recall	0.38 ± 0.36	0.14 ± 0.23
	F1 score	0.53 ± 0.04	0.51 ± 0.02
	False-negative ratio	0.61 ± 0.36	0.86 ± 0.23
Lasso regression model	Accuracy	0.77 ± 0.19	0.82 ± 0.15
	AUC	0.68 ± 0.05	0.59 ± 0.02
	Precision	0.22 ± 0.14	0.04 ± 0.04
	Recall	0.41 ± 0.45	0.15 ± 0.17
	F1 score	0.50 ± 0.02	0.47 ± 0.06
	False-negative ratio	0.59 ± 0.45	0.85 ± 0.17
Artificial neural network	Accuracy	0.95 ± 0.02	0.90 ± 0.04
	AUC	0.92 ± 0.01	0.54 ± 0.10
	Precision	0.82 ± 0.14	0.13 ± 0.25
	Recall	0.58 ± 0.17	0.00 ± 0.00
	F1 score	0.81 ± 0.06	0.48 ± 0.01
	False-negative ratio	0.42 ± 0.17	0.99 ± 0.00
Random forest classifier	Accuracy	0.82 ± 0.04	0.87 ± 0.08
	AUC	0.78 ± 0.01	0.60 ± 0.05
	Precision	0.20 ± 0.06	0.08 ± 0.10
	Recall	0.44 ± 0.11	0.16 ± 0.20
	F1 score	0.58 ± 0.03	0.51 ± 0.03
	False-negative ratio	0.56 ± 0.11	0.84 ± 0.20
Extreme gradient boosting	Accuracy	0.91 ± 0.03	0.92 ± 0.05
	AUC	0.75 ± 0.02	0.57 ± 0.03
	Precision	0.34 ± 0.07	0.08 ± 0.07
	Recall	0.18 ± 0.04	0.03 ± 0.04
	F1 score	0.59 ± 0.03	0.50 ± 0.03
	False-negative ratio	0.82 ± 0.04	0.97 ± 0.04
Support vector machines	Accuracy	0.93 ± 0.04	0.88 ± 0.05
	AUC	0.91 ± 0.01	0.54 ± 0.02
	Precision	0.61 ± 0.14	0.03 ± 0.05
	Recall	0.70 ± 0.28	0.03 ± 0.04
	F1 score	0.79 ± 0.10	0.48 ± 0.01
	False-negative ratio	0.30 ± 0.28	0.97 ± 0.04

*AUC* Area under recall-precision curve

**Table 4:** Top predictor importance

Feature	Importance
Surgical procedure	0.1689
Emergency Surgery	0.1346
Renal function	0.0999
Indication	0.0969
Liver metastasis	0.0710
Leukocytosis	0.0582
Preoperative steroid use	0.0571
Technique (hand-sewn)	0.0476
Active smoking	0.0447
Prior abdominal surgery	0.0400

*BMI* Body mass index, *CCI* charlson comorbidity index

## Discussion

In a pilot study using data from four centers in Switzerland for colorectal surgery, we assess the feasibility of predicting AL accurately from tabular data using ML techniques. Our findings demonstrate that predicting AL to a certain extent is feasible and identifies the most important input variables, laying the basis for a more extensive international multicenter study.

Even though a plethora of studies have analyzed risk factors for AL, up to this day, no reliable clinical prediction model for AL has been established [11–25]. This study aims to prove whether using a machine learning algorithm is proficient to solve the classification problem into AL and non-AL (‘will my patient suffer from AL after colorectal surgery?’).

Our results are promising, showing the potential of methodologies associated with machine learning for a prospective study for predicting AL. Souwer et al. [32] published 2020 a systematic review of existing models predicting mortality and complications after colorectal and colorectal cancer surgery. Seven models with the endpoint of AL prediction build a score based on standard statistical methods and ML techniques with a wide range of included patients between 159 and 10392 [33–39]. The range of the performance of the AUC values was between 0.63 and 0.95 for the development cohort and 0.58 to 1.0 for the validation cohort. Our model’s performance lies within these findings with an AUC of 0.78, respectively. Most of the studies used a combination of preoperative, intraoperative, and postoperative features, like duration of surgery [34–36], blood loss [36, 38], or wound infection [35] and, moreover, non-patient or procedural-related features, like hospital size [33]. We favored using predictors of the preoperative setting to aid in patient information prior to surgery.

A comparison to existing risk-calculating morbidity models, like the POSSUM score [40] or the ACS-NSQIP [41], should be done cautiously since the definition of complications and their severity differs. Moreover, the choice of different risk factors included and the lack of external validation makes a comparison challenging. Still, these findings will help develop a more precise future model. In general, predictive models and their performance are subject to their individual training and validation cohort and developing conditions, like regional and technical differences, risk profile of a population, and surgical indication. Therefore, validation or re-calibration with patient cohorts from different countries or hospital sizes would make them more sustainable and generalizable. Additionally, a re-calibration could be sensible after several years due to possible minor adaptions in current surgical practice.

The Random Forest model excels at providing probability-based predictions that are invaluable for informed clinical decision-making, particularly in an in-domain context. Similarly, the Logistic Regression model is adept at offering robust probability estimates that can be critical for decision-making in out-of-domain settings. While the model’s performance on the external holdout test set was notably lower in the Random Forest model, we attribute this discrepancy to a domain shift. If the robustness for out-of-domain generalization were prioritized, the Logistic Regression model would be the model of choice due to its more consistent performance. Given the pilot nature of this study, our focus is on assessing the model’s efficacy within a specific domain with the Random Forest model, understanding its capabilities and limitations, before considering external domain applications with the Logistic Regression model. It is crucial to emphasize that both models, due to their probabilistic nature, do not provide direct class label predictions but instead offer the probability associated with each instance, facilitating nuanced clinical judgment. Well-calibrated predicted probabilities are arguably more important in clinical practice (“How likely is it that I am going to experience AL?’—‘Your probability is 17%’) instead of binary predictions (‘Am I going to suffer from AL?’—‘The model predicts yes/no’). Physicians are experts at dealing with uncertainty and risks, and probabilities are thus more appreciated by patients and physicians than a mere yes or no answer—apart from the fact that patients are never binary but instead represent a spectrum of risk [42]. A rule-in model could prove to be of great value for clinicians by simply identifying the high-risk group, and, if possible, modifiable risk factors can be adjusted. Still, a model that is proficient to detect gray-zone patients at low risk for AL is of great value to identify those patients who conversely could be waived of protective stoma, thus, if an AL occurs might suffer from more severe complications. Nevertheless, our model is valuable for shared decision-making.

Clinical prediction models can facilitate assessing individual risks and making more informed decisions based on predictive analytics that are tailored to each patient. However, especially in colorectal surgery, the indication for surgery is rarely truly elective. Therefore, a prediction model can only help decide whether an intervention should be postponed to improve the risk profile or, especially for emergency interventions, whether a patient would benefit from a diverting stoma to minimize and modify risk factors before re-joining the colon. On the other hand, a comprehensive predictive model may also increase a patient’s acceptance of the primary placement of a protective stoma. Thus, such a model could potentially also help to improve the physician-patient relationship through enhanced patient education.

There is a widespread misunderstanding that variable importance measures gleaned from clinical prediction models can discover correlations and causalities like explanatory modeling does (prediction versus explanation) [43]. Indeed, this common misconceptualization exists because predictive and explanatory modeling are often not as explicitly distinguished as attempted here in this study. Indeed, the interchangeable use of the concepts of *in-sample correlation* and *out-of-sample generalization* can lead to false clinical decision-making [44]. While those variables identified as having high feature importance in this study may indeed be the most crucial ones for precise and generalizable prediction of AL, it cannot safely be concluded that these variables are necessarily also important independent risk factors for AL in their own right.

Another separate question is the initial choice of input variables for clinical prediction modeling, which can be achieved in various ways [45]. In any case, a balance between performance through the inclusion of many variables and between the goal of arriving at parsimonious models that truly generalize needs to be struck. The choice of variables for this study was focused on common risk factors described in literature and preoperatively available patient-related risk factors to minimize the statistical noise from differing standard procedures in distinct clinical centers.

Another difficulty in clinical prediction modeling is choosing the appropriate sample size. According to a common rule of thumb, there should be at least ten minority class observations in a dataset per feature [46]. This study relies on 21 patient-related risk factors, thus making a total number of 1000 patients with AL who would be necessary for training the final model. Other architectures, such as random forests, and SVMs seem to require much more data per feature [47]. Therefore, it is conceivable that including more patients will further refine the current model. Additionally, with more data, more complex methods can be implemented that would avoid the use of techniques such as data augmentation to generate synthetic information and further improve the model performance.

Yet, the results of a predictive model cannot be seen as a clear recommendation pro or contra an intervention as the risk profile is tailored only to a specific endpoint and thus does not entirely reflect the patient’s global situation. Indeed, components of decision-making such as the psychological distress of a patient with chronic diverticulitis are not included in the model and have a decisive influence on the indication. Consequently, prediction models should be seen only as adjunctive information to be used in a complementary way for informed shared decision-making. Nevertheless, the necessity for evidence-based clinical prediction models becomes clear when considering the relative inability of even experienced clinicians in predicting clinical outcomes [26], while the ethical implications of an ‘artificial intelligence doctor’ technology independent from human control have to be taken into account, too [48]. Consequently, ML-based clinical prediction models could be deemed a contemporary optimal trade-off between the clinical experience of human experts and the exploitation of big data by learning algorithms.

## Limitations

Besides the caveat of the retrospective data collection, our cohort’s relatively high AL rate of 9.0% can be seen as a limitation. Similarly, the difference in AL incidence among the dataset represents an additional hurdle that is realistic, as AL rate is described inconsistently in the literature [4, 6–10]. The patient population at the included hospitals with 23.8% emergency cases and a cohort that includes transplanted and immunosuppressed patients is expected to have higher complication rates [22]. Nevertheless, such a difference to other hospitals should be reflected in the ASA score, the CCI, and blood values and thus also in our results. By including patient data from other institutions in future analyses, this number will be balanced out, and a differentiated breakdown according to emergency interventions, immunosuppressant use, previous radio/chemotherapy, and cancer diagnosis, which additionally reflect a patient’s health status, is conceivable and could be implemented in our ML algorithm.

Furthermore, despite choosing common preoperative risk factors for AL from previous works, there are certainly several more unknown features influencing anastomotic healing we did not consider in our analysis. For instance, intraoperative factors like the surgeon’s experience are reported to influence postoperative morbidity [49, 50]. Zarnescu et al. [50] recently presented their summary of risk factors of AL, distributing them into pre-, intra-, and postoperative risk factors for AL, of which there are some modifiable and others are not. Other than that there might be further influencing circumstances leading to an AL, which are less convenient to measure and hence to include in a future ML algorithm, like blood flow or tension on the anastomosis. Aligning with this, we have included a broad variance of indications in our algorithm rather than performing a subgroup analysis, reflecting the daily situation of colon surgery also in smaller hospitals. As expected, the type of surgical procedure was one of the main features for the model performance.There is the potential that the future ML model, using multicenter data, will perform differently, and some other features will be more relevant in the algorithm. Recruiting more patient data from other hospitals is crucial and will further allow for more detailed statistical models and subgroup analyses, especially for less common surgical indications. The current algorithm will require updating and re-calibration, and the performance will be re-evaluated [51].

One further caveat of any model is the danger of overfitting. In clinical prediction modeling, overfitting means that an algorithm adheres too strictly to the training data, especially its inherent variance and possible noise factors (e.g., noise generated by a hospital’s standardized procedures). With enough training, the algorithm will perform extremely well on the training data while losing its generalization capability toward new data from other centers. Indeed, it is not unlikely that this study might suffer from slight overfitting due to standardized hospital procedures. However, this weakness could be addressed by recruiting more patient data from other hospitals. Furthermore, it is important to highlight that the ROC-AUC metric is influenced by class distribution imbalance. Here, the F1 score to demonstrate the model’s robustness in both categories.

Lastly, as of the nature of a pilot study, assessing the feasibility of a method in a limited patient cohort is a caveat, not allowing to draw clinical implications from the results so far. The performance of the external validation cohort, using a fairly small sample size with a rare event to be predicted, will be considerably impaired by each error in the prediction. According to the central limit theorem, we expect an enhanced performance of the internal and external validation sets using more patient data, again emphasizing the cruciality of more patient data after conducting this pilot study. Therefore, we have purposefully not yet deployed the model for clinical application in, e.g., a web-app, as any small sample sized and not extended externally validated clinical prediction model is not yet recommended for clinical use [52].

## Conclusion

In this pilot study, we developed an ML-based prediction model for AL after colorectal surgery using ten patient-related risk factors associated with AL. However, it is crucial to include and externally validate the results on international multicenter data with larger sample sizes to develop a robust and generalizable model.
